# The first record of *Cucullia umbratica* (Lepidoptera: Noctuidae) from Japan

**DOI:** 10.3897/BDJ.7.e34197

**Published:** 2019-04-10

**Authors:** Soichiro Noguchi, Takahiro Ochiai

**Affiliations:** 1 Systematic Entomology, Graduate School of Agriculture, Hokkaido University, Sapporo, Japan Systematic Entomology, Graduate School of Agriculture, Hokkaido University Sapporo Japan; 2 Wood Chemistry and Chemical Biology, Graduate School of Agriculture, Hokkaido University, Sapporo, Japan Wood Chemistry and Chemical Biology, Graduate School of Agriculture, Hokkaido University Sapporo Japan

**Keywords:** COI, *
Cucullia
*, Genbank, Hokkaidō, Japan, Lepidoptera, Noctuidae

## Abstract

**Background:**

*Cucullia umbratica* is known from Europe (from Spain to southern Fennoscandia), Russia, Afghanistan, Turkestan and Mongolia, in the Palearctic. In addition, introduction of this species to Canada has been reported recently.

**New information:**

We report this species from Japan for the first time, from two locations at Hokkaidō. The earliest record is from 2015.

## Introduction

*Cucullia umbratica* (Linnaeus, 1758) is a fairly large species of moth, with a wingspan of 48–57 mm (Heath and Emmet 1983). Due to its peculiar appearance, this species is generally called the “shark” moth (Price 2017). The larvae of this species utilise a wide range of Asteraceae as host plants (Hampson 1906, Heath and Emmet 1983). Although this species is widely distributed throughout the Palearctic (Hampson 1906, Heath and Emmet 1983, Dubatolov and Matov 2009), it has not been recorded from Japan to date (Kishida 2011, Jinbo 2017). In the present paper, we report this species from Hokkaidō for the first time and this also represents the first record of this species from Japan.

## Materials and methods

### Specimens

Dried specimens were used. The genitalia were dissected and stained with safranin and Fast Blue. Genital structures were observed under a Nikon SMZ 745T microscope. Digital images of the specimens were captured using a Nikon D5600 digital camera body and a Tamron SP 90 mm F2.8 Di MACRO lens and genitalia images were captured using a Canon EOS 5D Mark IV camera body with a Canon EF 100 mm F2.8 *L* IS USM Macro lens. The field photo was taken with a Canon EOS 6D camera body with a Canon EF 100mm F2.8 Macro lens and a Canon Macro Ring Lite ML-3. The morphological terminology used herein generally follows that of Heath (1976).

### Molecular methods

DNA was extracted from the distal portion of the 8th abdominal segment and the entire sample was suspended in extraction buffer following manufacturer's protocols for the DNeasy Blood & Tissue Kit (Qiagen, Inc., Valencia, CA, U.S.A.). The specimen used for DNA extraction was deposited in the insect collection at the Hokkaido University Museum (SEHU), Sapporo, Japan. Polymerase chain reaction (PCR) amplification of cytochrome oxidase I (COI) was carried out using an Applied Biosystems 2720 Thermal Cycler (Applied Biosystems, Foster City, CA, USA) with a touchdown amplification programme: 2 minutes at 94^o^C, 10 s at 94^o^C, 10 s at 55–46^o^C, 105 s at 68^o^C, 36 cycles of 10 s at 94^o^C, 10 s at 45^o^C, 105 s at 68^o^C and a final extension step for 2 minutes at 72^o^C. A single fragment of 1536 bp was amplified from the PCR amplification using primer pair CO1-exF (5-ATCGCCTAAACTTCAGCCATT-3) and TL-N-3017 (5-CTTAAATCCATTGCACTAATCTGCCATA-3) (Scheffer et al. 2004). PCR products were visualised via 1% agarose gel electrophoresis with ethidium bromide to confirm amplification. PCR products were purified with innuPREP PCRpure Light Kit (analytikjena, Jena, Germany) according to the manufacturer’s instructions. Purified PCR products were quantified using a Nanodrop One (Nanodrop Technologies, Wilmington, DE, USA). Purified products were sequenced in both directions using BigDye Terminator v3.1 chemistry (Applied Biosystems, Foster City, CA, USA). Primers used for sequencing were six primer sets (Table 1). The sequencing products were purified on Sephadex G–50 Fine DNA Grade (GE Healthcare, Chicago, U.S.A.) and analysed in 3500xL Genetic Analyzer (Applied Biosystems, Foster City, CA, USA).

### Sequence Analysis

Sequence contigs were assembled using BioLign Version 4.0.6.2 (2005; http://en.bio-soft.net/dna/BioLign.html) and aligned consensus sequences were created using BioEdit 7.0.5.3 (Hall 1999). The p–distance was calculated by comparing with 13 COI sequences registered in GenBank (accession numbers: KJ389042, GU686885, GU686845, KX043158, JF415531, GU828701, JF415532, JF415530, GU654999, KX040689, KM573523, HQ563405 and KJ183425) using MEGA (Molecular Evolutionary Genetics Analysis) Version 7.0 (Kumar et al. 2016).

## Taxon treatments

### Cucullia umbratica

Linnaeus, 1758

#### Materials

- **Type status:** Other material. **Occurrence:** recordedBy: Takuya Ito; individualCount: 1; sex: male; lifeStage: adult; **Taxon:** scientificName: Cucullia umbratica; kingdom: Animalia; phylum: Euarthropoda; class: Insecta; order: Lepidoptera; family: Noctuidae; genus: Cucullia; specificEpithet: umbratica; scientificNameAuthorship: Linnaeus; **Location:** country: Japan; stateProvince: Hokkaidō; county: Sōya-gun; municipality: Sarufutsu Village; locality: Hama-sarufutsu; **Identification:** identifiedBy: Takuya Ito; dateIdentified: 2015; **Event:** samplingProtocol: light trap; eventDate: 2015-07-18
- **Type status:** Other material. **Occurrence:** catalogNumber: 87764; recordedBy: Takuya Ito; individualCount: 1; sex: male; lifeStage: adult; **Taxon:** scientificName: Cucullia umbratica; kingdom: Animalia; phylum: Euarthropoda; class: Insecta; order: Lepidoptera; family: Noctuidae; genus: Cucullia; specificEpithet: umbratica; scientificNameAuthorship: Linnaeus; **Location:** country: Japan; stateProvince: Hokkaidō; county: Sōya-gun; municipality: Sarufutsu Village; locality: Hama-sarufutsu; **Identification:** identifiedBy: Takuya Ito; dateIdentified: 2015; **Event:** samplingProtocol: light trap; eventDate: 2015-08-01; fieldNotes: DNA extraction; **Record Level:** institutionCode: SEHU; collectionCode: Insect
- **Type status:** Other material. **Occurrence:** catalogNumber: 87761; recordedBy: Takuya Ito; individualCount: 1; sex: male; lifeStage: adult; **Taxon:** scientificName: Cucullia umbratica; kingdom: Animalia; phylum: Euarthropoda; class: Insecta; order: Lepidoptera; family: Noctuidae; genus: Cucullia; specificEpithet: umbratica; scientificNameAuthorship: Linnaeus; **Location:** country: Japan; stateProvince: Hokkaidō; county: Sōya-gun; municipality: Sarufutsu Village; locality: Hama-sarufutsu; **Identification:** identifiedBy: Takuya Ito; dateIdentified: 2016; **Event:** samplingProtocol: light trap; eventDate: 2016-07-23; fieldNotes: DNA extraction, DDBJ: LC460439; **Record Level:** institutionCode: SEHU; collectionCode: Insect
- **Type status:** Other material. **Occurrence:** recordedBy: Takahiro Ochiai; individualCount: 1; sex: male; lifeStage: adult; **Taxon:** scientificName: Cucullia umbratica; kingdom: Animalia; phylum: Euarthropoda; class: Insecta; order: Lepidoptera; family: Noctuidae; genus: Cucullia; specificEpithet: umbratica; scientificNameAuthorship: Linnaeus; **Location:** country: Japan; stateProvince: Hokkaidō; county: Sōya-gun; municipality: Sarufutsu Village; locality: Hama-sarufutsu; **Identification:** identifiedBy: Takuya Ito; dateIdentified: 2016; **Event:** samplingProtocol: light trap; eventDate: 2016-07-23
- **Type status:** Other material. **Occurrence:** recordedBy: Takahiro Ochiai; individualCount: 1; sex: male; lifeStage: adult; **Taxon:** scientificName: Cucullia umbratica; kingdom: Animalia; phylum: Euarthropoda; class: Insecta; order: Lepidoptera; family: Noctuidae; genus: Cucullia; specificEpithet: umbratica; scientificNameAuthorship: Linnaeus; **Location:** country: Japan; stateProvince: Hokkaidō; county: Sōya-gun; municipality: Sarufutsu Village; locality: Hama-sarufutsu; **Identification:** identifiedBy: Takuya Ito; dateIdentified: 2016; **Event:** samplingProtocol: light trap; eventDate: 2016-07-23
- **Type status:** Other material. **Occurrence:** recordedBy: Takuya Ito; individualCount: 1; sex: male; lifeStage: adult; **Taxon:** scientificName: Cucullia umbratica; kingdom: Animalia; phylum: Euarthropoda; class: Insecta; order: Lepidoptera; family: Noctuidae; genus: Cucullia; specificEpithet: umbratica; scientificNameAuthorship: Linnaeus; **Location:** country: Japan; stateProvince: Hokkaidō; county: Sōya-gun; municipality: Sarufutsu Village; locality: Hama-sarufutsu; **Identification:** identifiedBy: Takuya Ito; dateIdentified: 2017; **Event:** samplingProtocol: light trap; eventDate: 2017-07-22
- **Type status:** Other material. **Occurrence:** recordedBy: Takahiro Ochiai; individualCount: 1; sex: male; lifeStage: adult; **Taxon:** scientificName: Cucullia umbratica; kingdom: Animalia; phylum: Euarthropoda; class: Insecta; order: Lepidoptera; family: Noctuidae; genus: Cucullia; specificEpithet: umbratica; scientificNameAuthorship: Linnaeus; **Location:** country: Japan; stateProvince: Hokkaidō; county: Sōya-gun; municipality: Sarufutsu Village; locality: Hama-sarufutsu; **Identification:** identifiedBy: Takuya Ito; dateIdentified: 2017; **Event:** samplingProtocol: light trap; eventDate: 2017-07-22
- **Type status:** Other material. **Occurrence:** catalogNumber: 87763; recordedBy: Soichiro Noguchi; individualCount: 1; sex: male; lifeStage: adult; **Taxon:** scientificName: Cucullia umbratica; kingdom: Animalia; phylum: Euarthropoda; class: Insecta; order: Lepidoptera; family: Noctuidae; genus: Cucullia; specificEpithet: umbratica; scientificNameAuthorship: Linnaeus; **Location:** country: Japan; stateProvince: Hokkaidō; county: Sōya-gun; municipality: Sarufutsu Village; locality: Hama-sarufutsu; **Identification:** identifiedBy: Takuya Ito; dateIdentified: 2017; **Event:** samplingProtocol: light trap; eventDate: 2017-07-22; fieldNotes: DNA extraction; **Record Level:** institutionCode: SEHU; collectionCode: Insect
- **Type status:** Other material. **Occurrence:** catalogNumber: 87762; recordedBy: Soichiro Noguchi; individualCount: 1; sex: female; lifeStage: adult; **Taxon:** scientificName: Cucullia umbratica; kingdom: Animalia; phylum: Euarthropoda; class: Insecta; order: Lepidoptera; family: Noctuidae; genus: Cucullia; specificEpithet: umbratica; scientificNameAuthorship: Linnaeus; **Location:** country: Japan; stateProvince: Hokkaidō; county: Esashi-gun; municipality: Hamatonbetsu Town; locality: Aza-usotan; **Identification:** identifiedBy: Takuya Ito; dateIdentified: 2017; **Event:** samplingProtocol: light trap; eventDate: 2017-07-22; fieldNotes: DNA extraction; **Record Level:** institutionCode: SEHU; collectionCode: Insect
- **Type status:** Other material. **Occurrence:** recordedBy: Takuya Ito; individualCount: 1; sex: male; lifeStage: adult; **Taxon:** scientificName: Cucullia umbratica; kingdom: Animalia; phylum: Euarthropoda; class: Insecta; order: Lepidoptera; family: Noctuidae; genus: Cucullia; specificEpithet: umbratica; scientificNameAuthorship: Linnaeus; **Location:** country: Japan; stateProvince: Hokkaidō; county: Sōya-gun; municipality: Sarufutsu Village; locality: Hama-sarufutsu; **Identification:** identifiedBy: Takuya Ito; dateIdentified: 2017; **Event:** samplingProtocol: light trap; eventDate: 2017-07-29
- **Type status:** Other material. **Occurrence:** recordedBy: Takahiro Ochiai; individualCount: 1; sex: male; lifeStage: adult; **Taxon:** scientificName: Cucullia umbratica; kingdom: Animalia; phylum: Euarthropoda; class: Insecta; order: Lepidoptera; family: Noctuidae; genus: Cucullia; specificEpithet: umbratica; scientificNameAuthorship: Linnaeus; **Location:** country: Japan; stateProvince: Hokkaidō; county: Sōya-gun; municipality: Sarufutsu Village; locality: Hama-sarufutsu; **Identification:** identifiedBy: Takuya Ito; dateIdentified: 2018; **Event:** samplingProtocol: light trap; eventDate: 2018-07-28
- **Type status:** Other material. **Occurrence:** recordedBy: Soichiro Noguchi; individualCount: 1; sex: male; lifeStage: adult; **Taxon:** scientificName: Cucullia umbratica; kingdom: Animalia; phylum: Euarthropoda; class: Insecta; order: Lepidoptera; family: Noctuidae; genus: Cucullia; specificEpithet: umbratica; scientificNameAuthorship: Linnaeus; **Location:** country: Japan; stateProvince: Hokkaidō; county: Sōya-gun; municipality: Sarufutsu Village; locality: Hama-sarufutsu; **Identification:** identifiedBy: Takuya Ito; dateIdentified: 2018; **Event:** samplingProtocol: light trap; eventDate: 2018-07-28
- **Type status:** Other material. **Occurrence:** recordedBy: Takahiro Ochiai; individualCount: 1; sex: male; lifeStage: adult; **Taxon:** scientificName: Cucullia umbratica; kingdom: Animalia; phylum: Euarthropoda; class: Insecta; order: Lepidoptera; family: Noctuidae; genus: Cucullia; specificEpithet: umbratica; scientificNameAuthorship: Linnaeus; **Location:** country: Japan; stateProvince: Hokkaidō; county: Esashi-gun; municipality: Hamatonbetsu Town; locality: Aza-usotan; **Identification:** identifiedBy: Takuya Ito; dateIdentified: 2018; **Event:** samplingProtocol: light trap; eventDate: 2018-07-28

#### Diagnosis

**Figure 2.**
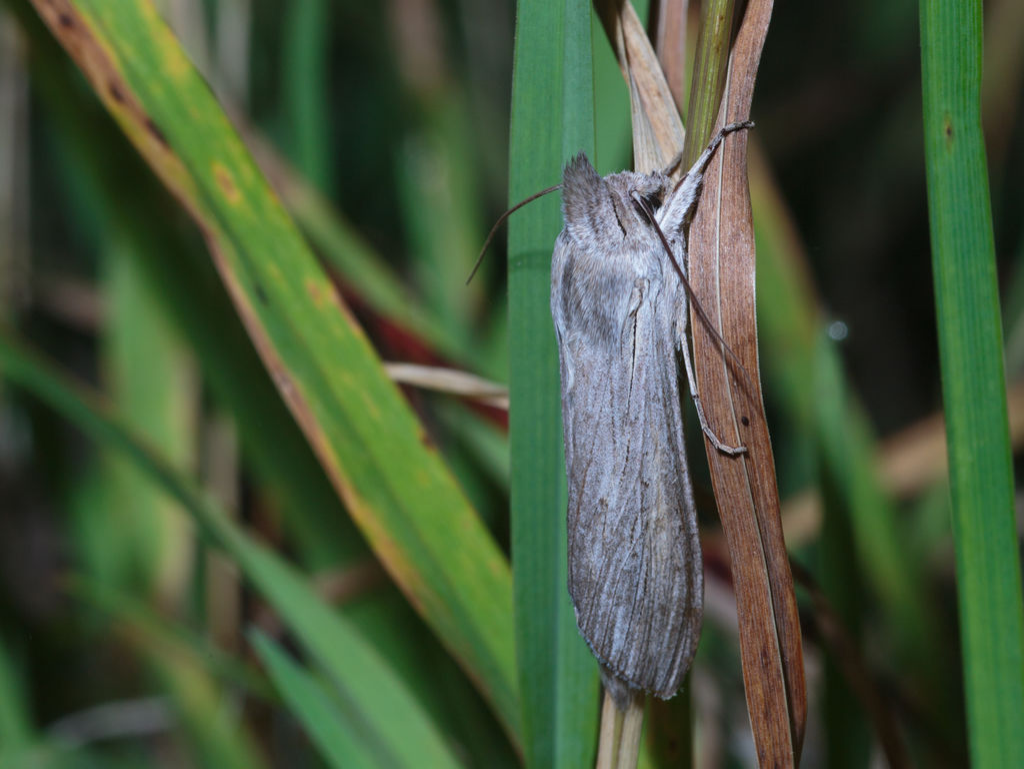
*Cucullia umbratica*, in Hama-sarufutsu, Sarufutsu-village, Sōya-gun, Hokkaidō, Japan. Photo by Takahiro Ochiai.

*Cucullia umbratica* can be distinguished from congeners by a combination of the following characters (Figs 1, 2): wingspan 48–57 mm; head and thorax grey mixed with brown; antenna with dorso-basally white; abdomen grey or brown; patagium elongate and forming a thoracic cowl; forewing ashen grey, the veins with dark brown; reniform and orbicular stigmata almost obsolete; basal steak to one–third of forewing, black and fine; terminal fascia dark brown but incomplete; the veins not extending into cilia; cilia white; hindwing white, the veins with brown but the latter sometimes reduced, in female the whole terminal half of hindwing brown; cilia white; terminal shade white with broken dark dividing line (Hampson 1906, Heath and Emmet 1983).

#### Distribution

Europe (from Spain to southern Fennoscandia), Russia, Afghanistan, Turkestan, Mongolia, North America (Hampson 1906, Heath and Emmet 1983, Dubatolov and Matov 2009, Handfield and Handfield 2010) and Japan (present study).

## Discussion

We have collected *Cucullia umbratica* every year in Sarufutsu village since 2015 and in Hamatonbetsu-chō since 2017. Therefore, this species is considered to be established at least around the Sarufutsu and Hamatonbetsu areas. We have collected 12 males and one female since 2015. These specimens have a wingspan of 47.5–53 mm, these sizes agreeing with Heath and Emmet (1983). Other characters also agree with the species diagnosis as shown in Hampson (1906), Heath and Emmet (1983). Especially, the genital structures are in complete agreement with those shown in Heath and Emmet (1983), Handfield and Handfield (2010). From the combination of the general and genital morphology (Fig. 3), we identified the species we collected as *Cucullia umbratica*.

We also sequenced the entire COI gene (1536 bp) of an individual collected at Hama-sarufutsu, Sarufutsu village, Sōya-gun, Hokkaidō, Japan, 23 July 2016, which was registered at DDBJ under the accession number LC460439. The preliminary comparisons showed that the sequence obtained from Hokkaidō is very close to the other registered COI sequences of *Cucullia umbratica*, collected from Europe (p-distances 0.000–0.005), Canada (0.002) and China (0.033) (Table 2). Although the COI sequence obtained from the Japanese sample was almost identical with that obtained from European and Canadian samples (the latter is thought to have been introduced from Europe: Handfield and Handfield 2010), p–distance between Japanese and Chinese samples was significantly high. If this genetic difference corresponds to the genetic differentiation between European and Asian populations of *Cucullia umbratica*, the present result provides support for the possibility that the Japanese population of this moth was introduced from Europe. However, the genetic distance, greater than 3% between European and Chinese populations, is far beyond the range of generally recognised intraspecific variations of Lepidoptera (Hebert et al. 2004) and the basis of the species identification for the Chinese specimen was not provided (Jin et al. 2018). Therefore, it might be possible that the COI barcode of Chinese *Cucullia umbratica* (Genbank accession number KJ183425) has been obtained from a misidentified specimen. Further research is necessary in the future to elucidate the intraspecific genetic structure of *Cucullia umbratica* and the origin of the Japanese population.

## Supplementary Material

XML Treatment for Cucullia umbratica

## Figures and Tables

**Figure 1a.**
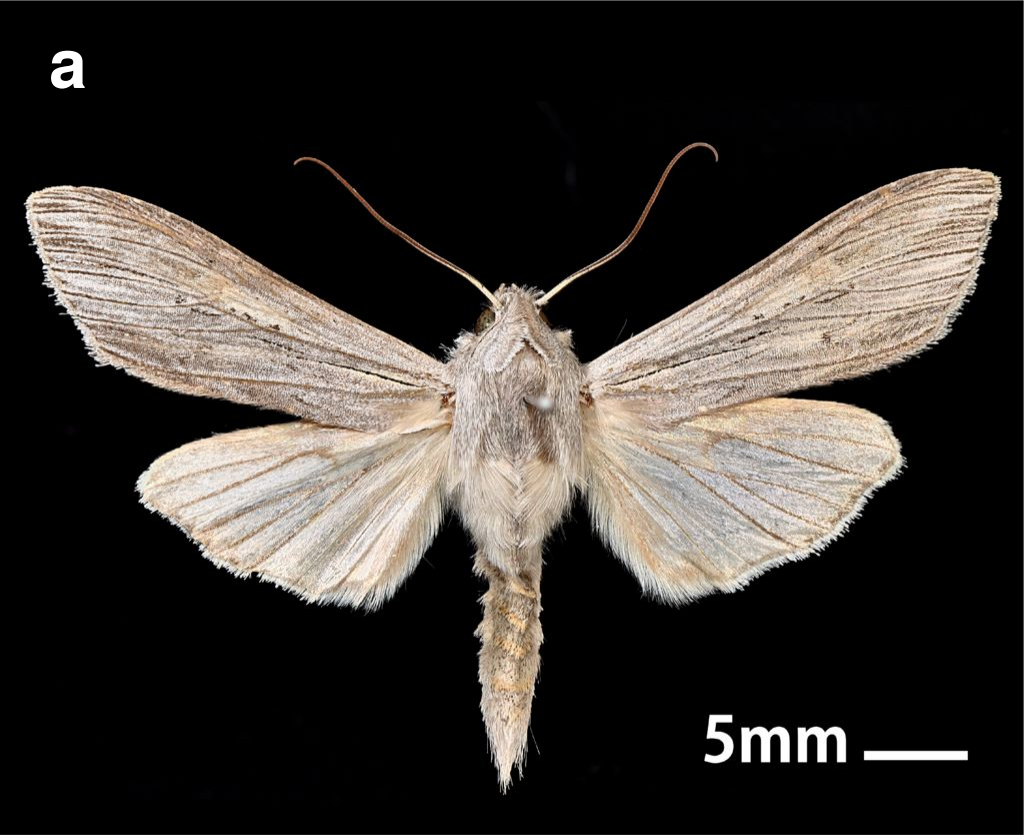


**Figure 1b.**
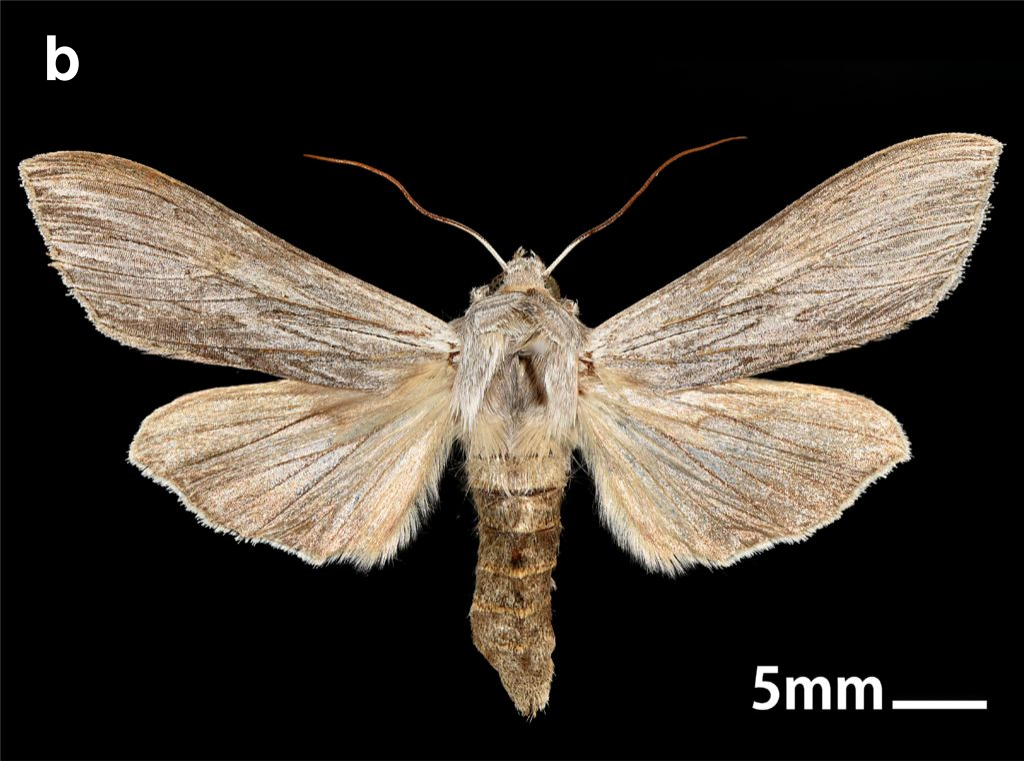


**Figure 3a.**
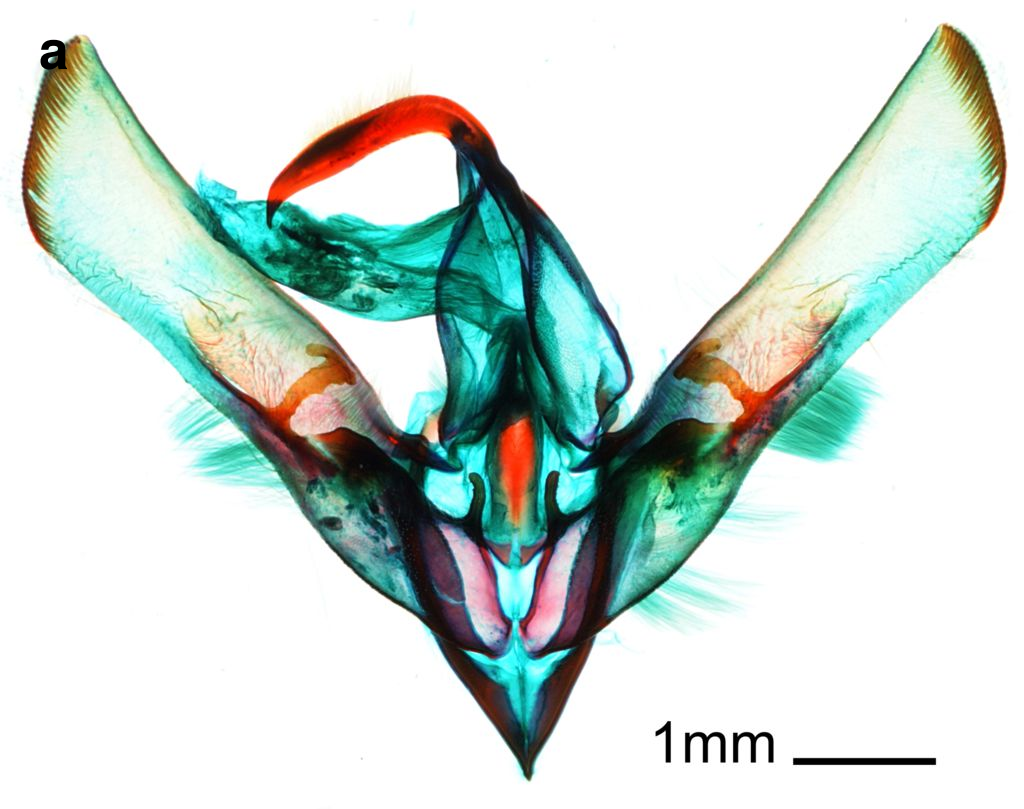
male genitalia, ventral view with aedeagus removed.

**Figure 3b.**
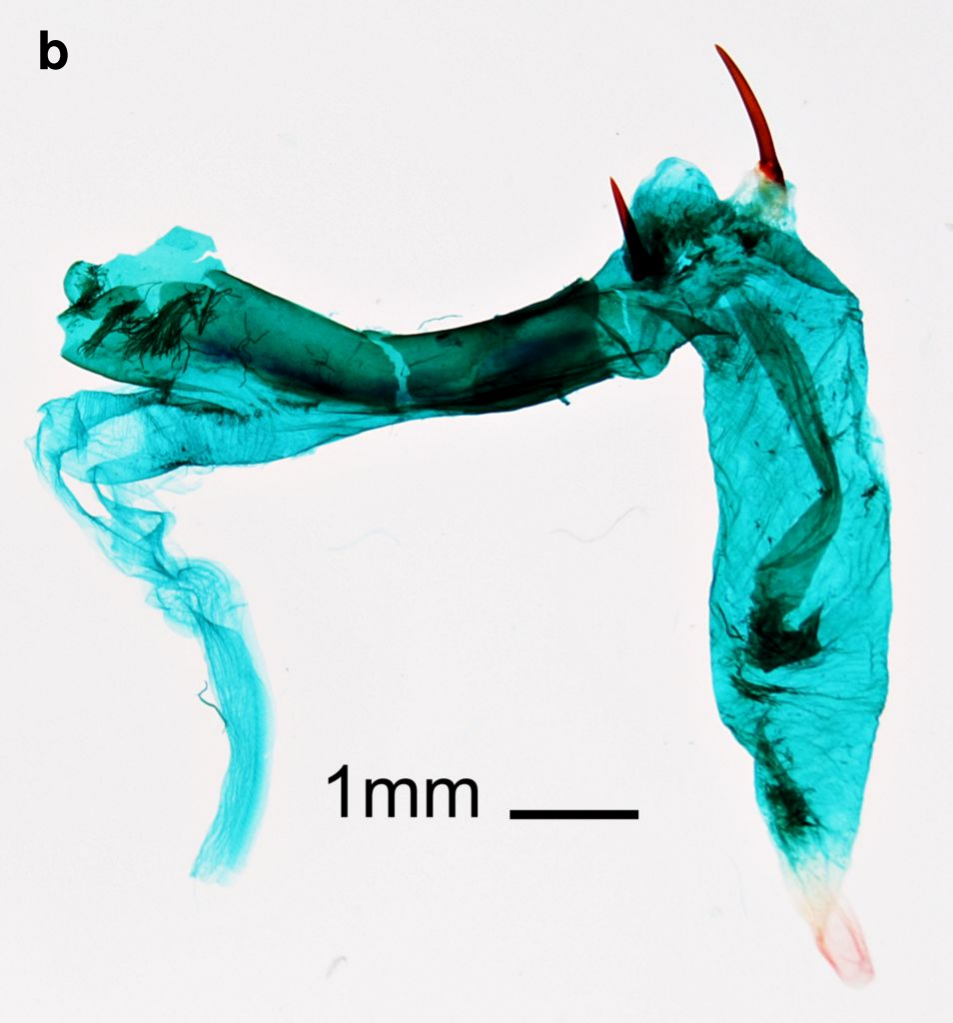
aedeagus with vesica everted.

**Table 1.** Primers used for sequencing

Primer name	Sequence (5'–3')	Source
CO1–exF	ATCGCCTAAACTTCAGCCATT	Present study
CO1–GK.1R	ACTGCACCTAAAATTGATGA	Present study
CO1–Hp.1F	AGCTGGAACAGGATGAAC	Present study
CO1–Hp.3R	TAGCAAAAACAGCTCCTA	Present study
CO1–Hp.3F	CTCTTCATGATACTTATTATG	Present study
TL–N–3017	CTTAAATCCATTGCACTAATCTGCCATA	Scheffer et al. 2004

**Table 2.** p–distances

Acc. Number	1	2	3	4	5	6	7	8	9	10	11	12	13	14
1. Present study														
2. KJ389042	0.002													
3. GU686885	0.005	0.004												
4. GU686845	0.002	0.000	0.004											
5. KX043158	0.002	0.000	0.004	0.000										
6. JF415531	0.002	0.000	0.004	0.000	0.000									
7. GU830689	0.004	0.002	0.005	0.002	0.002	0.002								
8. JF415532	0.002	0.000	0.004	0.000	0.000	0.000	0.002							
9. JF415530	0.002	0.000	0.004	0.000	0.000	0.000	0.002	0.000						
10. GU654999	0.002	0.000	0.004	0.000	0.000	0.000	0.002	0.000	0.000					
11. KX040689	0.002	0.000	0.004	0.000	0.000	0.000	0.002	0.000	0.000	0.000				
12. KM573523	0.002	0.000	0.004	0.000	0.000	0.000	0.002	0.000	0.000	0.000	0.000			
13. HQ563405	0.000	0.002	0.005	0.002	0.002	0.002	0.004	0.002	0.002	0.002	0.002	0.002		
14. KJ183425	0.033	0.035	0.035	0.035	0.035	0.035	0.033	0.035	0.035	0.035	0.035	0.035	0.033	
